# BDE-47 Induces Immunotoxicity in RAW264.7 Macrophages through the Reactive Oxygen Species-Mediated Mitochondrial Apoptotic Pathway

**DOI:** 10.3390/molecules28052036

**Published:** 2023-02-21

**Authors:** Qian Gao, Zhong-Yuan Zhou, Ya-Ning He, Ming-Hui Dong, Zhao-Ning Wang, Hong-Mei Chen

**Affiliations:** 1 Key Laboratory of Xinjiang Phytomedicine Resources and Utilization, Ministry of Education, School of Pharmacy, Shihezi University, Shihezi 832002, China; 2 Pharmacology Department, School of Pharmacy, Shihezi University, Shihezi 832002, China; 3 Department of Marine Ecology, College of Marine Life Science, Ocean University of China, Qingdao 266003, China

**Keywords:** PBDE, immunotoxicity, macrophage, reactive oxygen species, apoptosis, immune function

## Abstract

Polybrominated diphenyl ethers (PBDEs) are classic and emerging pollutants that are potentially harmful to the human immune system. Research on their immunotoxicity and mechanisms suggests that they play an important role in the resulting pernicious effects of PBDEs. 2,2′,4,4′-Tetrabrominated biphenyl ether (BDE-47) is the most biotoxic PBDE congener, and, in this study, we evaluated its toxicity toward RAW264.7 cells of mouse macrophages. The results show that exposure to BDE-47 led to a significant decrease in cell viability and a prominent increase in apoptosis. A decrease in mitochondrial membrane potential (MMP) and an increase in cytochrome C release and caspase cascade activation thus demonstrate that cell apoptosis induced by BDE-47 occurs via the mitochondrial pathway. In addition, BDE-47 inhibits phagocytosis in RAW264.7 cells, changes the related immune factor index, and causes immune function damage. Furthermore, we discovered a significant increase in the level of cellular reactive oxygen species (ROS), and the regulation of genes linked to oxidative stress was also demonstrated using transcriptome sequencing. The degree of apoptosis and immune function impairment caused by BDE-47 could be reversed after treatment with the antioxidant NAC and, conversely, exacerbated by treatment with the ROS-inducer BSO. These findings indicate that oxidative damage caused by BDE-47 is a critical event that leads to mitochondrial apoptosis in RAW264.7 macrophages, ultimately resulting in the suppression of immune function.

## 1. Introduction

As a kind of flame retardant, polybrominated diphenyl ethers (PBDEs) are widely utilized in both residential and commercial items [1,2]. They are highly fat soluble and difficult to break down. They can be transported in diverse environmental media following discharge into the environment [3] and reach the human body following the bioconcentration and amplification effect of the food chain [4]. In 2004, analysts noted that the total amount of PBDEs in human blood, milk, and tissue had increased by nearly 100 times in the past three decades [5]. Due to their toxicity and persistence, some PBDEs were restricted by the Stockholm Convention in 2009 and 2017 [6], although the use of PBDEs has been considerably reduced in recent years. However, affected by electronics recycling and time lag effects, PBDEs in the environment will continue to be detected in the coming decades [7,8]. From 2000 to 2019, a meta-analysis of the global distribution and trend of PBDEs in human blood and milk indicated no significant declining trend [9]. It is nevertheless critical to pay attention to the research on the toxicity of PBDEs and other chemicals, particularly 2,2′,4,4′-tetrabrominated biphenyl ether (BDE-47) and 2,2′,4,4′,5-pentabromodiphenyl ether (BDE-99), which are relatively more hazardous to human health and the environment [10].

Many studies have recently revealed the systemic toxic effect of PBDEs on reproduction [11], nerves [12,13], and development [14,15]. PBDEs also damage target organs such as the liver [16,17] and kidneys [18]. The immune system is an important defense system of biological organisms, and functional changes in this system can serve as a sensitive index for evaluating the safety of exogenous chemicals. Although data on PBDEs immunotoxicity are limited, some studies on rodents have shown that long-term exposure to PBDEs damages components of the immune systems of mice and rats [19,20,21,22]. In studies of other animals, young salmon exposed to PBDEs (270 ng/g) in the lower sections of the Willamette River demonstrated higher susceptibility to illness, which may be connected to immune function impairment [23]. Environmentally realistic concentrations (0−634 ng/g wet diet) of PBDEs are able to alter the immune function of frogs [24], and similar results were presented for in vitro toxicity research. Exposure to some brominated flame retardants (DE-71, DE-79, etc.) in a concentration range of 0.01–10 mg/mL for 24 h may stimulate primary splenocytes by boosting antigen-presenting-related molecule expression and IL-4 production [25]. Finally, concentrations above 0.01 μg/mL of DE-71 can enhance the production of normal human PBMC cytokines stimulated by LPS or PHA-L in vitro [26]. Exposure to BDE-47 at concentrations above 5 μM or BDE-209 at concentrations above 20 μM for 24 h can increase intracellular reactive oxygen species (ROS) formation, activate apoptosis pathways, and damage immune function, such as cell phagocytosis, in peritoneal macrophages [27]. PBDEs have features of low bioaccessibility [28], easy compound adsorption onto plastic [29], and binding to serum proteins [30], meaning that not all PBDEs can enter the cells [31]. Thus, the concentration of PBDEs examined in experiments is higher than the environmental concentration. In addition, the effect of PBDEs is influenced by the concentration of exposure, wherein higher concentrations that exceed the defense ability can cause toxic damage. Depending on the concentration, PBDEs can either stimulate or inhibit the autoimmune response of various immune cells to some extent. Different immune response indicators and response trends also complicate the investigation of immunotoxicity mechanisms and the development of effective defense pathways.

Research on immunotoxicity mechanisms currently focuses on mitochondrial apoptosis and the control of ROS. Apoptosis mediated by the mitochondrial pathway is frequently triggered by ROS, which causes structural changes, rupture, and fragmentation of the mitochondrial membrane system, with subsequent programmed cell death [32]. ROS and nitric oxide (NO) generated by cell oxidative stress can disrupt redox equilibrium, thus resulting in damage to proteins, DNA, and membranes, and finally promoting cell death [33]. Ivermectin (IVM) has previously been demonstrated to induce the overproduction of macrophage ROS, cytochrome C release, and damage to double-stranded DNA, indicating that it might cause immunotoxicity via immunological malfunction and cytotoxicity [34]. Exposure to microplastics and two persistent organic contaminants can considerably affect immune responses in clams and dramatically increase their intracellular ROS levels [35]. The immune response induced by the fungicides azoxystrobin (AZO) and iprodione (IPR) can be partially reversed by the antioxidant N-acetylcysteine (NAC) [36]. Similar findings have been shown in related studies of PBDE immunotoxicity in recent years. When seals were exposed to PBDE homologs at a higher concentration, the ROS levels and phagocytosis were affected [37].

Macrophages are a type of immune cell that is used to study cell phagocytosis, cellular immunity, and molecular immunology. They are essential for the host to defend itself against foreign substances. On the one hand, macrophages can induce inflammation via the use of reactive nitrogen (RNS) and ROS receptors to mediate phagocytosis and particle clearance, activating intracellular inflammatory bodies, caspase processes, and the release of inflammatory mediators [38]. On the other hand, they present antigens by expressing co-stimulatory molecules and specific cytokines, thereby initiating and controlling specific T cell responses. As a result, macrophages are critical for both the innate and adaptive immune systems [39]. The RAW264.7 cell line is an immortal mouse macrophage line that is frequently used as an in vitro model to study the immunotoxicity of environmental pollutants. RAW264.7 cells were chosen to investigate the immunotoxicity and potential mechanisms of BDE-47 in this study, with a focus on the mitochondrial apoptotic pathway and phagocytosis, antigen presentation, inflammatory response, phenotypic activation, and other immune responses. Furthermore, the antioxidant N-acetyl-L-cysteine (NAC) and ROS-inducer L-buthionine-(S,R)-sulfoximine(BSO) were applied to study the correlation between immunotoxicity and intracellular ROS levels induced by BDE-47 in RAW264.7 cells.

## 2. Results

### 2.1. Effects of BDE-47 on Cell Viability and Morphology

**Figure 1:**
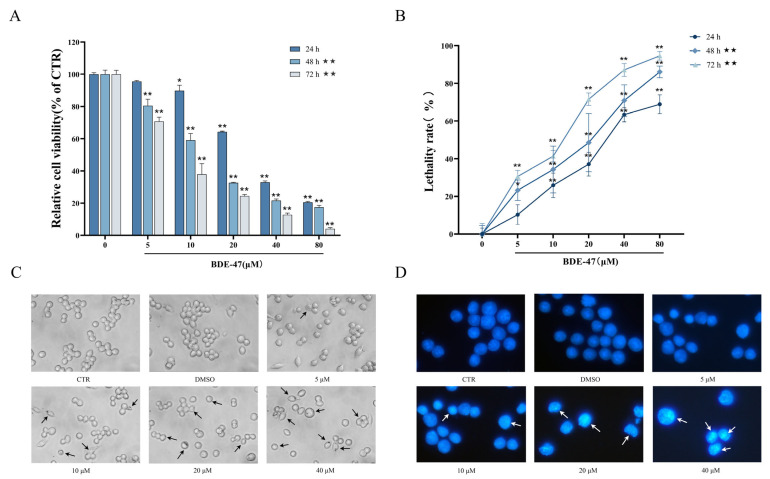
Effect of BDE-47 on proliferative viability and morphology in RAW264.7 cells. (**A**) Cell proliferation viability characterized by MTT assay (*n* = 6). (**B**) Cell mortality determined using trypan blue staining (*n* = 6). (**C**) Cells photographed under light microscopy (200×). Arrows indicate cells exhibiting cell shrinkage and cytoplasmic membrane blebbing. (**D**) Cells stained with DAPI and photographed under inverted fluorescence microscopy (400×). Arrows indicate condensed nuclei. * Significant difference compared with the control (* *p* < 0.05; ** *p* < 0.01). ★ Significant difference compared with BDE-47-treated 6 h group (★★ *p* < 0.01).

The results of an MTT assay showed that, with an increase in BDE-47 concentration, the proliferation viability of RAW264.7 cells presented a trend of decreasing with increasing time and concentration (Figure 1A). This difference was significant at concentrations above 10 μM at 24 h (*p* < 0.05), and, when beyond 48 h, the results exhibited a very significant difference for concentrations above 5 μM (*p* < 0.01). The rate of cell lethality also exhibited time and concentration-dependent increases (Figure 1B). The cell lethality rate at 24 h markedly increased in the groups treated with concentrations above 10 μM (*p* < 0.01). The obtained results are consistent with the findings of the MTT assay.

The number of cells decreased with the increase in the concentration of BDE-47 used in the treatment for 24 h (Figure 1C). For normal RAW264.7 cells, the edges are clear, the refractive index of the cells is higher, and a small number of cells become spindle-shaped in the DMSO solvent control and 5 μM BDE-47 groups. At concentrations greater than 10 μM, the number of cells is significantly reduced, and the cell edges appear blurry. When the concentration of BDE-47 is 40 μM, the microscopic cells become blurred and black, and a large number of cells are broken. When analyzed by DAPI nuclear staining, treatment with BDE-47 results in the nuclei becoming brighter, solidified, and concentrated, and partially fragmented forms appeared compared with the CTR group (Figure 1D).

### 2.2. BDE-47-Induced Apoptosis in RAW264.7 Cells Is Mediated by the Mitochondrial Pathway

**Figure 2:**
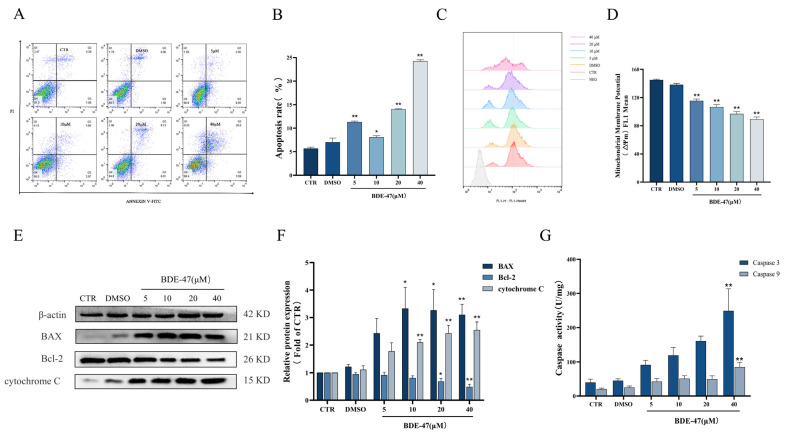
Effect of BDE-47 on apoptosis in RAW264.7 via the mitochondrial pathway. (**A**,**B**) Representative graphs and quantitative data of cell apoptosis obtained from flow cytometry analysis. (**C**,**D**) Representative graphs and quantification of mean fluorescence value of MMP changes obtained from flow cytometry analysis. (**E**,**F**) Protein expression level and quantitative statistics of BAX, Bcl-2, and cytochrome C. (**G**) Changes in Caspase-3 and Caspase-9 enzyme activities. *n* = 3; * *p* < 0.01; ** *p* < 0.05.

The BDE-47-induced RAW264.7 cell apoptosis in a concentration-dependent manner. The cell apoptotic rate increased steadily (from 11.34 ± 0.12% to 24.26 ± 0.17%) after 24 h exposure, and the difference between the treated groups and the control group was statistically significant (*p* < 0.05) (Figure 2A,B). The intensity of rhodamine 123 fluorescence in the cells gradually decreased with the increase in the BDE-47 concentration, which indicates a drop in MMP levels. A significant decrease in the MMP levels was observed for 5, 10, 20, and 40 μM treatments compared with the control (*p* < 0.01). For instance, the intensity of rhodamine 123 fluorescence decreased to 38.41 ± 1.40% in the treated group of 40 μM at 24 h, which was much lower than that of the CTR group (Figure 2C,D). According to the Western blot results, the expression of pro-apoptotic protein BAX in RAW274.7 cells exposed to BDE-47 showed an increasing trend. The expression level of anti-apoptotic protein Bcl-2 showed the opposite trend, with its expression level significantly reduced after treatment with BDE-47 at concentrations above 20 μM (*p* < 0.01). The protein expression of cytochrome C was also examined in cells; it increased in the cytoplasm after BDE-47 exposure, and the differences werE significant at concentrations above 10 μM (*p* < 0.01) (Figure 2E,F). The results indicate that cytochrome C is released from the mitochondria into the cytoplasm. No significant changes in the activities of Caspase-3 and Caspase-9 enzymes were observed between the solvent control group and the control group, while their activities showed an increasing trend with the increase in the BDE-47 concentration, which was significant in the 40 μM treatment group (*p* < 0.01) (Figure 2G).

### 2.3. BDE-47 Alters Immune Function of RAW264.7 Cells

**Figure 3:**
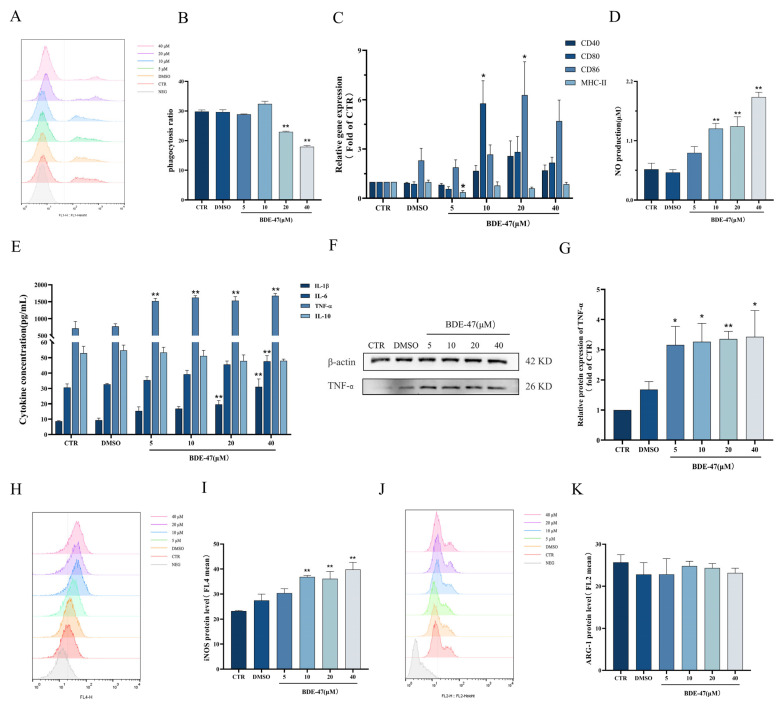
Effect of BDE-47 on immune function and polarization phenotype of RAW264.7 cells. (**A**,**B**) Representative graphs and quantitative data of cell phagocytosis ratios obtained from flow cytometry analysis. (**C**) Gene expression levels of presented antigens. (**D**) NO release level. (**E**) Levels of IL-1β, IL-6, TNF-α, and IL-10 secretion. (**F**,**G**) Protein expression level and quantitative statistics of TNF-α. (**H**,**I**) Representative graphs and quantitative data of iNOS expression levels obtained from flow cytometry analysis. (**J**,**K**) Representative graphs and quantitative data of Arg-1 expression levels obtained from flow cytometry analysis. *n* = 3; * *p* < 0.01, ** *p* < 0.05.

The capacity of RAW264.7 cells to phagocytize *E. coli* gradually decreases with the treatment of BDE-47 for 24 h, and this was significant for the 20 and 40 μM treatments compared with the CTR (*p* < 0.05) (Figure 3A,B). BDE-47 treatment could decrease the gene expression of exogenous antigen-presenting molecule MCH-II, which was significant for the 5 μM treatment (*p* < 0.05). The gene expression levels of co-stimulators CD40, CD80, and CD86 were significantly increased after exposure to BDE-47. The expression level of the CD80 gene was significantly increased at 10 μM (*p* < 0.05), and the expression level of CD86 was obviously increased at 20 μM (*p* < 0.05) (Figure 3C). Moreover, NO production was steadily elevated with the increase in the BDE-47 concentration, being significant at concentrations greater than 10 μM compared with the CTR group (Figure 3D). BDE-47 also increased the secretion of inflammatory factors IL-1β, IL-6, and TNF-α. Compared with the CTR group, the cytokine level of IL-6 was greatly increased, and significance was observed at a concentration of 40 μM (*p* < 0.01); IL-1β was significantly elevated at 20 and 40 μM (*p* < 0.05). The cytokine levels of TNF-α were greatly elevated with the increase in the BDE-47 concentration compared with the CTR group (*p* < 0.01). However, there was no significant change in the anti-inflammatory factor IL-10 after exposure to BDE-47 (Figure 3E). We further examined the level of intracellular TNF-α protein expression using Western blotting experiments. The results are shown in Figure 3F,G. After BDE-47 exposure, the expression level of TNF-α protein in RAW264.7 cells was significantly increased. The markers of different phenotypes of RAW264.7 macrophages were detected by flow cytometry. The expression level of iNOS, which is an M1 pro-inflammatory phenotype marker, was elevated with the increase in the BDE-47 concentration (Figure 3H,I). However, the expression level of M2 anti-inflammatory phenotypic marker Arg-1 exhibited no significant change compared with the CTR group after BDE-47 exposure (Figure 3J,K).

### 2.4. BDE-47 Increases the Level of Reactive Oxygen Species in RAW264.7 Cells and the Regulation of Oxidative Stress Genes

**Figure 4:**
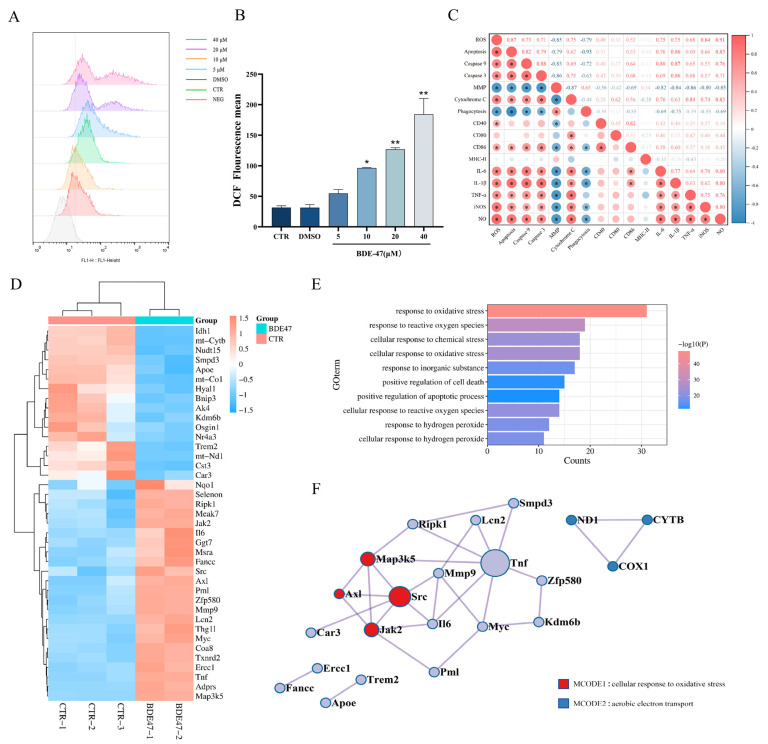
Effect of BDE-47 treatment on intracellular level of ROS and oxidative stress regulatory network in RAW264.7 cells. (**A**,**B**) Representative graphs and quantitative data of ROS levels obtained from flow cytometry analysis after BDE-47 treatment. (**C**) Pearson correlation analysis of the relevant metrics. (**D**) Differentially expressed genes associated with oxidative stress in transcriptome sequencing. (**E**) Biological process analysis of genes differentially expressed under oxidative stress. (**F**) Protein–protein network interaction analysis of differentially expressed oxidative stress genes. *n* = 3; * *p* < 0.01, ** *p* < 0.05.

DCFH-DA was used to detect the intracellular ROS levels, and the results show a trend of concentration-dependent increases with the BDE-47 exposure. A significant increase was observed for the concentrations greater than 10 μM BDE-47 (*p* < 0.05) (Figure 4A,B). In the Pearson correlation matrix, a strong negative correlation was observed between ROS levels and cell proliferative viability, MMP, and cell phagocytosis capacity (r = 0.8–1, *p* < 0.01). However, a strong positive correlation was observed between ROS levels and cell mortality, apoptotic rate, Caspase-3 and Caspase-9 enzyme activity, cytochrome C protein expression, CD40, CD86, IL-1β, IL-6, TNF-α, and iNOS gene expression, and the capacity to generate NO (Figure 4C). The 440 oxidative stress genes obtained in the CTD database were mapped to the differentially expressed genes (1113 upregulated, 696 downregulated) obtained by transcriptome sequencing, yielding 39 genes differentially regulated by oxidative stress, of which 23 are upregulated and 16 are downregulated (Figure 4D). The biological processes found, by Metascape analysis, to be enriched were mainly related to “response to oxidative stress response”, “response to reactive oxygen species”, and “positive regulation of apoptotic process” (Figure 4E). The protein–protein interaction network is shown in Figure 4F. The modular analysis identified two core MCODEs, a cellular response to oxidative stress, and aerobic electron transport. Increased ROS and modulation of genes associated with oxidative stress in the transcriptome provide further evidence that ROS is a critical regulator of BDE-47 stress in RAW264.7 cells.

### 2.5. Role of ROS in BDE-47-Induced Apoptosis and Immune Impairment in RAW264.7 Cells

#### 2.5.1. Effect of Antioxidant NAC Pretreatment on BDE-47-Induced Apoptosis and Cellular Immune Function on RAW264.7 Cells

**Figure 5:**
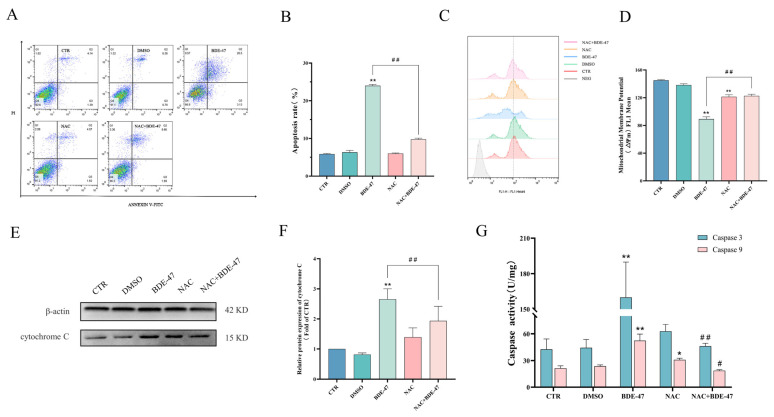
Effect of NAC pretreatment on BDE-47-induced apoptosis in RAW264.7 cells. Cells were pre-incubated with or without 100 μM NAC and then treated with BDE-47. (**A**,**B**) Cell apoptosis is shown in flow cytometric graphs and histogram. (**C**,**D**) Changes in MMP are shown in flow cytometric graphs and histogram. (**E**,**F**) Protein expression level and quantitative statistics of cytochrome C. (**G**) Changes in Caspase-3 and Caspase-9 enzyme activities. *n* = 3; * Significant difference compared with the untreated controls (* *p* < 0.05; ** *p* < 0.01). # Significant difference compared with the BDE-47-treated group (# *p* < 0.05; ## *p* < 0.01).

The antioxidant NAC was employed in the pretreatment to more thoroughly characterize the role of ROS in the process of stress induced by BDE-47 on RAW264.7. The results indicate that NAC could reduce the rate of BDE-47-induced apoptosis, while there was no significant effect on the apoptotic rate due to NAC treatment alone. Analyses of the apoptotic rate revealed that treatment with a combination of NAC and BDE-47 reduced the apoptotic rate (from 24.00 ± 0.20% to 9.76 ± 0.18%) compared with BDE-47 alone (Figure 5A,B). Moreover, the NAC measurably alleviated the effects of BDE-47, causing a drop in MMP and an increase in the intensity of rhodamine 123 fluorescence (from 38.41 ± 1.40% to 15.39 ± 1.04%) compared with BDE-47 only (Figure 5C,D). BDE-47 caused an elevation in the levels of cytochrome C protein in the cytoplasm, which was inhibited by the NAC. Treatment with NAC in combination with BDE-47 decreased the expression of cytochrome C compared with the BDE-47 group (*p* < 0.01) (Figure 5E,F). The increase in Caspase-3 and Caspase-9 enzyme activities due to BDE-47 were also inhibited by the NAC (Figure 5G).

**Figure 6:**
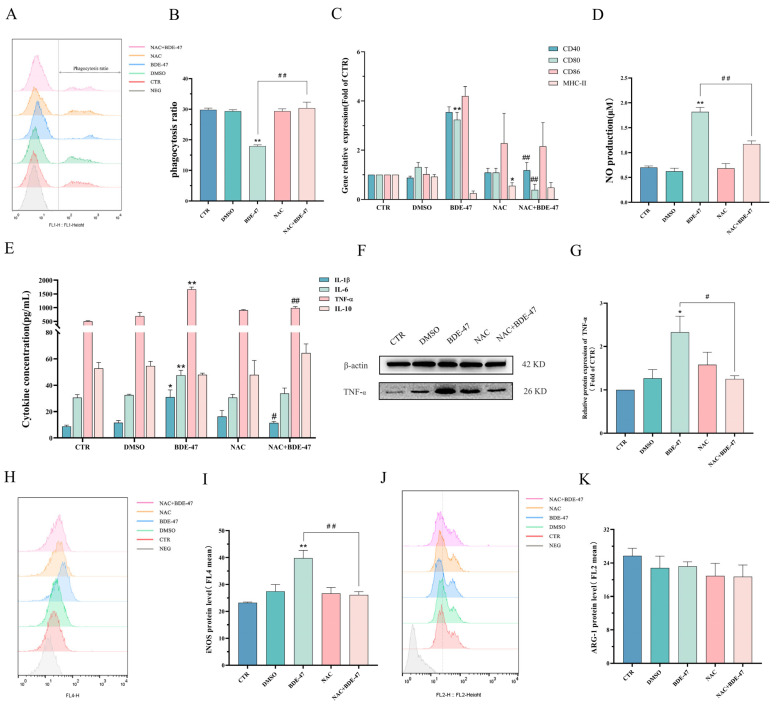
Effect of NAC pretreatment on immune function and polarization phenotype after BDE-47 exposure of RAW264.7 cells. Cells were pre-incubated with or without 100 μM NAC and then treated with BDE-47. (**A**,**B**) Cell phagocytosis function shown in flow cytometric graphs and histogram. (**C**) Gene expression levels of antigen presentation. (**D**) NO release levels. (**E**) Levels of IL-1β, IL-6, TNF-α, and IL-10 secretion. (**F**,**G**) Protein expression level and quantitative statistics of TNF-α. Flow cytometric graphs and histogram of (**H**,**I**) iNOS, and (**J**,**K**) Arg-1 protein levels. *n* = 3. * Significant difference compared with the untreated controls (* *p* < 0.05; ** *p* < 0.01). # Significant difference compared with the BDE-47-treated group (# *p* < 0.05; ## *p* < 0.01).

NAC-alleviated BDE-47 caused a decrease in phagocytosis capacity. The capacity oF phagocytosis increased (from 17.93 ± 0.25% to 30.37 ± 1.12%) compared with the BDE-47 treatment (Figure 6A,B). BDE-47 caused elevated levels of CD40, CD80, IL-1β, and TNF-α, which were inhibited by the NAC pretreatment (*p* < 0.05). Similar results were obtained in Western blotting experiments, where NAC pretreatment reduced the degree of increase in TNF-α protein expression caused by BDE-47 exposure (Figure 6F,G). Furthermore, the NAC and BDE-47 combined-treatment group showed a lower protein expression of NO release level and iNOS protein level compared with the BDE-47 group (*p* < 0.01). The effect of NAC pretreatment on the expression of CD86, IL-6, MHC-II, IL-10, and Arg-1 had no significant change after the BDE-47 exposure (Figure 6C–E,H–K).

#### 2.5.2. Effect of Inducer BSO Pretreatment on Apoptosis and Cellular Immune Function after BDE-47 Exposure

**Figure 7:**
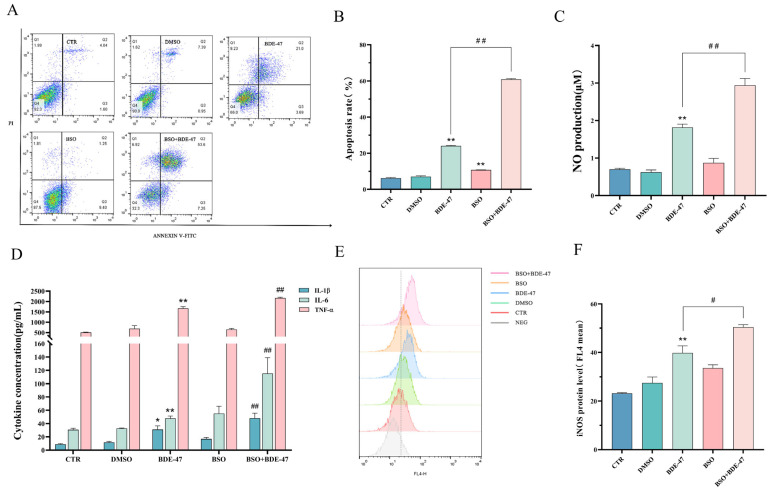
Effect of BSO pretreatment on apoptosis and immune function after BDE-47 exposure of RAW264.7 cells. Cells were pre-incubated, with or without 500 μM BSO, and then treated with BDE-47. (**A**,**B**) Cell apoptosis is shown in flow cytometric graphs and histogram. (**C**) NO release levels. (**D**) Levels of IL-1β, IL-6, and TNF-α secretion. (**E**,**F**) Expression of iNOS protein level is shown in flow cytometric graphs and histogram. *n* = 3. * Significant difference compared with the untreated controls (* *p* < 0.05; ** *p* < 0.01). # Significant difference compared with the BDE-47-treated group (# *p* < 0.05; ## *p* < 0.01).

BSO pretreatment increased the rates of BDE-47-induced apoptosis of RAW264.7 cells from 24.00 ± 0.20% to 60.86 ± 0.27% (Figure 7A,B), and the degree of apoptosis was also intensified, with a significance level of *p* < 0.01. In addition, we also measured the level of NO production and inflammatory factor secretion after the BSO pretreatment, and the results (Figure 7C,D) were the same as those for the BDE-47-treated group. BSO pretreatment leads to NO release and increased levels of secretion of the inflammatory factors IL-1β, IL-6, and TNF-α (*p* < 0.01). Finally, we used flow cytometry to detect the macrophage M1-type marker, iNOS, at the protein level, revealing that pretreatment with the inducer BSO enables increased polarization of RAW264.7 M1 pro-inflammatory typing after BDE-47 induction (*p* < 0.05).

## 3. Discussion

Exposure to BDE-47 can affect the immune system, manifesting as susceptibility to diseases in the body, abnormal immune responses, immune dysfunction, and cellular damage [27,40,41]. Macrophages have an innate immune defense mechanism and are crucial to many pathophysiological processes. This type of immune cell is found in many tissues of the human body, characterized by its powerful phagocytic activity, and it is highly active and sensitive. In this study, we report for the first time that BDE-47 can significantly damage the activity and immune function of RAW264.7 mouse macrophages in vitro, and we explore the role of ROS in immunotoxicity caused by BDE-47.

In the present study, a concentration range of 5 to 80 μM BDE-47 was chosen to investigate cell toxicity, according to our preliminary study and other previous studies [42,43]. With the increase in concentration, the cell viability was significantly reduced for 24, 48, and 72 h. However, the cell mortality increased with the increase in BDE-47 concentration, indicating that BDE-47 inhibits the proliferation of RAW264.7 mouse macrophages. Similar results were observed in other cells exposed to BDE-47 [16,44,45,46,47,48,49]. BDE-47-induced immunotoxicity is attributed to a decrease in the number of macrophages in various tissues in the body and weakened proliferative activity, which interferes with responses of the immune system, similar to the perfluorinated compounds PFOA and PFOS [50].

Current research on the immunotoxicity mechanism of exogenous compounds is mainly focused on autophagy [51], apoptosis [27], oxidative damage [52], and inflammatory response [53]. Mitochondrial pathway-mediated apoptosis is a process of cell death that is highly regulated by apoptosis genes. In our findings, the protein expression of Bcl-2 decreased and BAX significantly increased. Furthermore, the MMP levels decreased, cytochrome C release to the cytoplasm was increased, and the enzyme activities of Caspase-3 and Caspase-9 were upregulated, suggesting that the endogenous mitochondrial apoptotic pathway was triggered. These phenomena are consistent with our previous studies on Neuro-2a cells [13]. There was also a similar result in a study on acrylamide, which was found to induce caspase-dependent apoptosis in mouse splenocytes through mitochondrial-dependent signaling [54].

Macrophages play an extremely important role in inflammation, an innate immune response [55], and they exert defensive functions through phenotypic polarization, cytokines release, and phagocytosis of foreign particles to maintain the stability of the internal environment [38]. To further characterize the damage to the macrophage immune function resulting from BDE-47, we examined changes in phagocytosis, antigen presentation, cytokines secretion levels, and phenotypic markers. The results show that BDE-47 reduces the phagocytic capacity of macrophages and the expression of antigen-presenting molecules but increases the expression of co-stimulating factors and the secretion of inflammatory factors. Macrophages are divided into at least two major polarized phenotypes: M1-polarized macrophages and M2-activated macrophages [56]. We found that BDE-47 significantly promotes macrophage polarization to type M1, manifested by the elevated expression of the pro-inflammatory cytokines TNF-α, IL-6, and IL-1β, increased expression of TNF-α protein in cells, and increased NO release levels, triggering an inflammatory response in the body that is accompanied by an elevated expression of the macrophage-type M1 polarization marker, iNOS. Nevertheless, BDE-47 has no significant effect on macrophage polarization to the M2 type. The above data show that BDE-47 can significantly affect the macrophage immune function, such as through decreased phagocytosis, blocking of antigen presentation, and increased secretion of inflammatory factors, promoting NO production. Moreover, BDE-47 also has a certain impact on the phenotype of macrophages, inducing macrophages to M1-type polarization. Bisphenol A, one of the most widely used industrial compounds for synthesizing materials such as polycarbonate (PC) and epoxy resin, can promote mouse intraperitoneal macrophage differentiation from the pro-inflammatory M1-subtype but inhibits differentiation from the anti-inflammatory M2-subtype macrophages [57]. PM2.5 in the air can significantly enhance the polarization of macrophage inflammation M1-type, interfering with the balance of macrophage inflammation M1 and anti-inflammatory M2 polarization [58]. The above findings are consistent with our results.

ROS is often considered an essential regulatory factor when macrophages defend against inflammation and other stress [59]. In our study, ROS levels significantly increased with the concentration of BDE-47 used in exposure to RAW264.7 cells. The ROS that accumulates in macrophages can not only serve as an upstream factor to trigger apoptosis [60] but also directly damage biological macromolecules, eliciting various inflammatory responses [61]. ROS plays a mediating role between innate and adaptive immune cells and further influences immune processes, such as T cell activation, by antigen delivery [62]. A Pearson correlation analysis of biological indicators shows a significant correlation with ROS levels in most cells (Figure 4C). The transcriptome sequencing results further supported our previous results, such as for IL-6; TNF upregulated oxidative stress-associated genes also showed the same trend in protein expression in our ELISA and Western blotting results. To further explore the effects of ROS on BDE-47-induced immunotoxicity, the antioxidant N-acetylcysteine (NAC) was utilized to study the relationship between the immunotoxicity and ROS level caused by BDE-47 in RAW264.7 cells. The cytotoxicity results show that pretreatment with NAC could attenuate BDE-47-caused MMP damage, cytochrome C release, and the increased enzyme activity of Caspase-3 and Caspase-9, eventually reversing apoptosis. The biological process enrichment and protein interaction network modular analysis of the differentially transcriptionally expressed oxidative stress genes revealed that they were closely related to apoptosis and the transfer of aerobic electron chains in mitochondria, which, to some extent, supports our research finding that ROS can regulate the mitochondrial apoptotic pathway.

For the immune function, NAC can inhibit a degree of decreased cell phagocytosis function after exposure to BDE-47. It has been demonstrated that an increase in ROS is linked with M1-type activation in macrophages [63], which further stimulates the release of pro-inflammatory cytokines. NAC also alleviates the production level of NO, the elevated secretion of inflammatory factors, gene expression of co-stimulating factors, and protein expression of TNF-α and the M1 macrophage marker, iNOS. NAC was found to mitigate immunotoxicity in other studies, where NAC pretreatment could alleviate the immunotoxic effects of azolinone and isomethoxamine fungicides in mice in vitro [36]. Hou et al. showed that the immunotoxicity of aflatoxin B1 and ochratoxin A is associated with ROS regulation, and the antioxidant NAC can mitigate the combined toxicity in porcine alveolar macrophages [64]. However, we also found that gene expression levels of the exogenous antigen-presenting complex, MHC-II, did not increase significantly after NAC pretreatment, and minimal correlation was also observed in the Pearson correlation analysis, suggesting that it may have little involvement in the regulation of ROS. More research is still required to explain the observed phenomena. In addition, we also used pretreatment with the ROS-inducer BSO to explore the effect of ROS elevation on BDE-47-induced immunotoxicity [65]. Compared with the NAC pretreatment, diametrically opposite trends are shown: after the BSO pretreatment, several key indicators, such as apoptosis, NO production, inflammatory factor level, and M1 polarization of RAW264.7 cells, were significantly increased. This indicates that the BDE-47-induced immunotoxicity was intensified.

**Figure 8:**
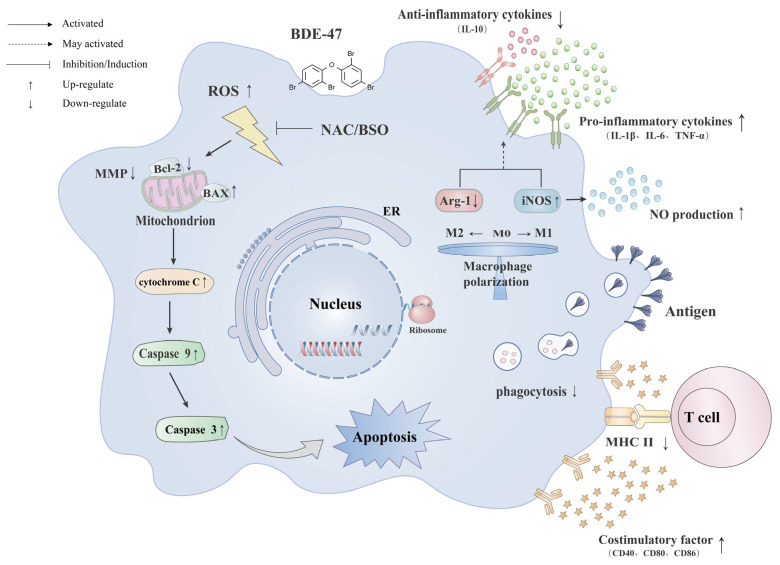
The mechanism of BDE-47-induced immunotoxicity in RAW264.7 cells.

Therefore, our results suggest that the environmental pollutant BDE-47 can cause an increase in intracellular ROS levels in macrophages. Genes related to oxidative stress showed transcriptional differences, and the intracellular antioxidant protection mechanism was affected by the increase in BDE-47 concentration. Moreover, BDE-47 further induces apoptosis of cells through the mitochondrial pathway, damaging immune function and generating immunotoxic effects on macrophages (Figure 8).

## 4. Materials and Methods

### 4.1. Chemicals and Reagents

BDE-47 was purchased from AccuStandard (New Haven, CT, USA). DCFH2-DA, thiazole blue, and dimethyl sulfoxide (DMSO) were purchased from Sigma (St. Louis, MO, USA). The Annexin V-FITC/PI apoptosis kit was purchased from BD Bioscience (Franklin Lake, NJ, USA). Arg-1 and iNOS antibodies were purchased from eBioscience (San Diego, CA, USA). *E. coli* (strain K-12) was purchased from Invitrogen (Carlsbad, CA, USA). The IL-10 enzyme-linked immunosorbent assay (ELISA) kit was purchased from MultiSciences (Hangzhou, China). Cytochrome C antibodies, IL-6, IL-1β, TNF-α ELISA kits, DAPI dye, rhodamine 123, and Caspase-3 and Caspase-9 activity assay kits were purchased from Beyotime Biotechnology (Shanghai, China). BAX, Bcl-2, and TNF-α antibodies were purchased from WanLeiBio Co., Ltd. (Shenyang, China). ECL luminescent reagent was purchased from Sangon Biotech Co., Ltd. (Shanghai, China). Real-time fluorescent quantitative reagents were purchased from Takara Biomedical Technology Co., Ltd. (Beijing, China). N-acetylcysteine (NAC) and L-buthionine-(S,R)-sulfoximine (BSO) were purchased from Yuanye Bio-Technology Co., Ltd. (Shanghai, China).

### 4.2. Cell Culture and Experimental Design

The RAW264.7 mouse macrophage line was obtained from the Shanghai Institute of Cell Biology (Chinese Academy of Sciences, Shanghai, China). The cells were cultured in DMEM (Gibco, Grand Island, NE, USA), containing 10% fetal bovine serum (Biological Industries, Kibbutz Beit Haemek, Israel) and 1% penicillin–streptomycin antibiotics (Gibco, Grand Island, NE, USA), and then cultured in an incubator at 37 °C and 5% CO_2_.

Logarithmically growing RAW264.7 cells were inoculated in 96-well and 6-well plates at a concentration of 2.5 × 10^4^ and 2.5 × 10^5^ mL^−1^, respectively, and cultured overnight. The cells were then treated with BDE-47 at various concentrations (0, 5, 10, 20, and 40 μM) for 24 h for the determination of cell viability, fluorescence level, protein, and mRNA expression. Untreated cells were used as the control (CTR); cells treated with 0.1% DMSO (*v*/*v*) were used as the DMSO solvent control (DMSO). In addition, RAW264.7 cells were precultured in 100 μM NAC or 500 μM BSO for 4 h, and then 40 μM of BDE-47 was added to the cells for 24 h, followed by the calculation of indexes related to apoptosis and immune function, and for studying the relationship between ROS and immunotoxicity.

### 4.3. Cytotoxicity Evaluation

The RAW264.7 cell proliferation activity was measured using the MTT assay. In brief, the cells were exposed to various concentrations of BDE-47 for 24, 48, and 72 h; then, 10 μL of MTT solution was added to each well and incubated at 37 °C for 4 h. After that, 150 μL DMSO was added to each well to dissolve the methylzan; then, a microplate reader was used to determine the absorbance (OD) value at 490 nm. Cell viability was calculated as the percentage of live cells relative to the total number of cells. Trypan blue staining was used to determine the rate of cell death, as in previous studies [66,67].

### 4.4. Morphology Observation

After the exposure to BDE-47, the cell surface morphological changes were directly visualized through an inverted microscope. Additionally, the cells were stained with 0.1 μg/mL DAPI staining solution for 30 min, followed by washing with PBS twice. Then, the cells were placed under a fluorescence microscope to observe cell nucleus pyknosis.

### 4.5. Apoptosis Detection

Cell apoptotic rates were detected using an Annexin V-FITC/PI staining kit. The cells were digested and collected following EDTA-free trypsinization and washed twice with pre-chilled PBS before being resuspended in Annexin-V binding buffer. The cells were stained with PI and Annexin V-FITC for 10 min in the dark. The stained cells were assessed using a BD flow cytometer, and FlowJo software was used to analyze the flow cytometry data. The apoptotic rate was expressed as the sum of the Q2 quadrant (the percentage of late apoptotic cells) and Q3 quadrant (the percentage of early apoptotic cells).

### 4.6. Detection of Mitochondrial Membrane Potential (MMP)

Rhodamine 123 chemical dye was used to evaluate the changes in the MMP. The treated cells were incubated with rhodamine 123 (5 mg/mL) for 30 min. The cells were collected by centrifugation and resuspended in PBS. The average FL1 channel fluorescence intensity, as assessed using the BD flow cytometer, was taken to represent the MMP in the cells.

### 4.7. Examination of ROS Level

Intracellular ROS formation was determined using the DCFH-DA probe. Briefly, after the exposure treatment of the cells, the supernatant was aspirated and washed before adding the DCFH2-DA probe (10 μM), then incubating for 30 min. The fluorescence intensity in the FL1 channel was then measured using the BD flow cytometer.

### 4.8. Caspase Enzymatic Activity

The Caspase-3 and Caspase-9 activities were determined using commercial Caspase-3 and Caspase-9 activity assay kits. First, the treated cell samples were collected, the protein extracted, and the concentration was then determined using the Bradford method. Subsequently, 50 μL of the sample was added to the assay buffer and catalytic substrate for reaction and incubated at 37 °C overnight. The absorbance at 405 nm was detected using a microplate reader, and the units of enzyme activity were calculated, according to the standard curve, as well as the sample protein concentration.

### 4.9. Western Blotting

The protein expression levels were determined by Western blot analysis, according to a previous study [68]. Briefly, protein samples were extracted using RIPA buffer, separated by SDS-PAGE, transferred to PVDF membranes, incubated for 12 h with antibodies diluted at the appropriate proportion (BAX antibody 1:1000; Bcl-2 antibody 1:1000; cytochrome C antibody: 1:200), and, for 1 h, with horseradish peroxidase-conjugated antibodies. The membrane was washed with TBST three times for 5 min each. The immunoreactive bands were detected with ECL reagents, according to the manufacturer’s instructions. ImageJ software was used to quantify the bands, and the protein expression levels of BAX, Bcl-2, and cytochrome C were standardized, based on comparison with β-actin protein.

### 4.10. Phagocytic Capacity

The treated cells were cultured in a serum-free fresh medium containing the biotinylated particles of FITC-labeled *Escherichia coli* (K-12 strain) for 1 h. After that, the cells were collected and washed three times with PBS to eliminate unincorporated *E. coli*, and the cell fluorescence was then detected by using flow cytometry, according to the method referred to in a previous study [69].

### 4.11. Quantitative Real-Time Polymerase Chain Reaction (QRT-PCR) Analysis

Total RNA was extracted from the treated RAW264.7 cells with TRIizol, according to the manufacturer’s protocol. Then, the cDNA was synthesized from the total RNA using a first-strand cDNA synthesis kit. The real-time PCR was performed using SYBR Green Supermix and PCR primers in a cycler. The sequences of primers used in the real-time PCR are listed in Table 1. The amplification was performed in 20 μL of the total mixture volume. The amplification program was as follows: predenaturation at 95 °C for 30 s, followed by 40 cycles of 95 °C for 5 s, and 60 °C for 34 s. GAPDH was used for normalization in the relative quantitative analysis, and the standard 2^−ΔΔCt^ method was used in the calculation.

### 4.12. Cytokine Secretion Levels and NO Production

The supernatant of the treated cells was collected, and the cytokine levels of IL-10, IL-6, IL-1β, and TNF-α were detected by ELISA, according to the manufacturer’s instructions. The NO production in each group was determined using the Griess kit, with the OD determined at 550 nm wavelength.

### 4.13. Cell Phenotype

INOS and Arg-1 are classic markers of the M1 pro-inflammatory phenotype and M2 anti-inflammatory phenotype, respectively. The detection of polarization marker protein expression levels was carried out using flow cytometry, according to a method referred to in previous studies [70,71]. The treated cells were fixed with a fixative solution for 20 min at 25 °C, and, after being washed with PBS twice, the cells were treated with a permeabilization solution and then incubated with PE-labeled Arg-1 and APC-labeled iNOS antibodies for 30 min at 4 °C in the dark. Then, the samples were analyzed by BD flow cytometry after washing.

### 4.14. RNA-Seq and Analysis of the Oxidative Stress Regulatory Network

RAW264.7 cells were treated with BDE-47 at a concentration of 20 μM (BDE-47) for 24 h and then harvested. The RNA extraction, purity, and quantification, and the transcriptome sequencing and analysis were performed by Personalbio Technology Co., Ltd. (Shanghai, China). After the sequencing, clean reads were obtained and aligned to the mouse genome, and the differentially expressed genes (DEGs) between the two groups were analyzed using DESeq (2012) R software. The significance of the gene expression differences was assessed with the *p*-value < 0.05 and log2 (fold change) > 1 as the threshold value. Relevant entries were retrieved from the CTD database (https://ctdbase.org, accessed on 15 October 2022) with the keyword “oxidative stress” to obtain associated genes, which were mapped to the differentially expressed genes obtained from the transcriptome sequencing. Gene ontology and protein–protein interaction analyses were performed using Metascape (https://metascape.org, accessed on 5 November 2022).

### 4.15. Data Analysis

For each experiment, we analyzed three or more replicates in a completely random manner. The experimental data are the average ± standard error (mean ± SEM), with GraphPad Prism 8.4 software for chart visualization. SPSS 26 software was used for the statistical analysis of the data. The HSD Tukey test was used to assess the significance between the control and experimental groups, and *p* < 0.05 indicates significance. Pearson correlation was used to analyze the interaction between the variables, for which visualization was realized using the ggplot2 package.

## 5. Conclusions

We found that BDE-47 can induce apoptosis of RAW264.7 cells via the mitochondrial pathway, causing immune function damage. The ROS inhibitor NAC could reverse apoptosis and alleviate the immune impairment caused by BDE-47, which could be conversely exacerbated by the inducer BSO. Intracellular ROS levels were found to be one of the regulators leading to BDE-47 immunotoxicity.

## Figures and Tables

**Table 1:** Specific primer sequences for *qRT-PCR*.

Target Gene	Forward (5′-3′)	Reverse (5′-3′)
MHC-II	GTGTGCAGACACAACTACGAGG	CTGTCACTGAGCAGACCAGAGT
CD40	ACCAGCAAGGATTGCGAGGCAT	GGATGACAGACGGTATCAGTGG
CD80	CCTCAAGTTTCCATGTCCAAGGC	GAGGAGAGTTGTAACGGCAAGG
CD86	ACGTATTGGAAGGAGATTACAGCT	TCTGTCAGCGTTACTATCCCGC
GAPDH	CATCACTGCCACCCAGAAGACTG	ATGCCAGTGAGCTTCCCGTTCAG

## Data Availability

The data are available on request due to privacy. The data presented in this study are available on request from the corresponding author.

## Sample Availability

Samples of the compounds are not available.
